# *ADAS*-viewer: web-based application for integrative analysis of multi-omics data in Alzheimer’s disease

**DOI:** 10.1038/s41540-021-00177-7

**Published:** 2021-03-19

**Authors:** Seonggyun Han, Jaehang Shin, Hyeim Jung, Jane Ryu, Habtamu Minassie, Kwangsik Nho, Insong Koh, Younghee Lee

**Affiliations:** 1 Department of Biomedical Informatics, University of Utah School of Medicine, Salt Lake City, UT USA; 2 Department of Biomedical Informatics, University of Utah Asia campus, Incheon, South Korea; 3 Center for Computational Biology and Bioinformatics, Indiana University School of Medicine, Indianapolis, IN USA; 4 Department of Radiology and Imaging Sciences and Indiana Alzheimer Disease Center, Indiana University School of Medicine, Indianapolis, IN USA; 5 Department of Physiology, Hanyang University, Seoul, South Korea

**Keywords:** Computational biology and bioinformatics, Neurology

## Abstract

Alzheimer’s disease (AD) is a neurodegenerative disorder and is represented by complicated biological mechanisms and complexity of brain tissue. Our understanding of the complicated molecular architecture that contributes to AD progression benefits from performing comprehensive and systemic investigations with multi-layered molecular and biological data from different brain regions. Since recently different independent studies generated various omics data in different brain regions of AD patients, multi-omics data integration can be a useful resource for better comprehensive understanding of AD. Here we present a web platform, *ADAS*-viewer, that provides researchers with the ability to comprehensively investigate and visualize multi-omics data from multiple brain regions of AD patients. *ADAS*-viewer offers means to identify functional changes in transcript and exon expression (i.e., alternative splicing) along with associated genetic or epigenetic regulatory effects. Specifically, it integrates genomic, transcriptomic, methylation, and miRNA data collected from seven different brain regions (cerebellum, temporal cortex, dorsolateral prefrontal cortex, frontal pole, inferior frontal gyrus, parahippocampal gyrus, and superior temporal gyrus) across three independent cohort datasets. *ADAS*-viewer is particularly useful as a web-based application for analyzing and visualizing multi-omics data across multiple brain regions at both transcript and exon level, allowing the identification of candidate biomarkers of Alzheimer’s disease.

## Introduction

Alzheimer’s disease (AD) is a neurodegenerative disorder rooted in complicated biological mechanisms and the inherent complexity of brain tissue^1,2^. Each area of the human brain has a unique function; for example, the parietal cortex is uniquely involved in the movement and incorporation of signals from other cortices^3,4^. This tissue-level complexity is reflected in transcriptomic and proteomic profiles of the brain, which feature dynamic and region-specific patterns across functionally different brain regions, including in the context of AD^5,6^. In particular, the regional specificity of molecular mechanisms in the brain means that neurodegenerative diseases may progress in a brain region-specific manner, and thus a genomic view of molecular status across multiple brain regions may be helpful for better understanding AD pathology.

Furthermore, not only is each type of omics profile (such as genomics, epigenomics, and transcriptomics) informative on its own, but integrated omics data can be useful for developing a broader molecular understanding of the mechanisms that underlie this complex disease^7^, improving our ability to elucidate the biological connections between gene expression and genetic regulation. For instance, extensive transcriptional alteration occurs during AD progression, with changes in alternative splicing (AS) resulting in remarkable changes in isoform expression^4,8–10^. In fact, the brain is among the areas that feature the greatest incidence of tissue-specific AS, and brain-specific AS patterns are known to be particularly important during neural development processes such as neuronal migration and axon guidance^4,9,10^. Many alternatively spliced genes have been identified in AD cases; in addition, GWAS studies have identified a large set of genetic variants associated with the onset and progression of AD^4,11,12^. Integration of these findings into multi-omics analyses is essential for the functional annotation of identified genetic variants.

In addition to genetic and transcriptomic data, epigenomic data from AD patients has also been made available, which allows for the integration of epigenetic elements such as methylation and miRNA expression with transcriptomic and genomic data. Epigenetic elements have been identified as potential biomarkers for AD^13–15^, with methylation in particular being tightly linked to transcript expression changes (i.e., alternative splicing as a mechanism of gene regulation)^16,17^. Therefore, there is both opportunity and need to develop an integrative presentation that brings together transcriptomic, genomic, and epigenomic data in order to gain insight into the molecular mechanisms at work across and within brain regions in AD.

Here, we present *ADAS*-viewer, a web-based statistical analysis platform with which researchers can explore multi-omics data across seven brain regions in the context of AD. *ADAS*-viewer features RNA-seq and WGS data collected from the cerebellum (CER), temporal cortex (TCX), dorsolateral prefrontal cortex (DLPFC), frontal pole (FP), inferior frontal gyrus (IFG), parahippocampal gyrus (PHG), and superior temporal gyrus (STG) in three independent patient cohorts of the Accelerating Medicines Partnership-Alzheimer’s Disease (AMP-AD) project (10.7303/syn2580853): the Mayo Clinic^18^, the ROS and MAP studies (ROSMAP)^19^, and the Mount Sinai Brain Bank (MSBB)^20^. The ROSMAP dataset includes cognitively normal old adults and AD patients; meanwhile, CER and TCX data were collected from cases having progressive supranuclear palsy (PSP) or pathologic aging. In addition, *ADAS*-viewer provides a statistical test for determining associations between expression and epigenetic data collected from the DLPFC region in the ROSMAP cohort, such as transcriptomic data versus methylation or miRNA data. Finally, by incorporating clinical information such as sex, ethnicity, diagnosis, plaque mean, and Braak stage, *ADAS*-viewer allows the user to conduct association tests between these phenotypes and the selected multi-omics data; for example, it can evaluate differences between AD and normal groups, sex-specific molecular traits in AD, differences between AD and dementia patients, or association of transcripts/AS exons with Braak stage. We present *ADAS*-viewer as having potential utility for identifying biomarkers and developing further understanding of AD pathology with respect to differential alternative splicing and the genetic/epigenetic regulation of gene expression (i.e., eQTL and sQTL) across seven brain regions.

## Results

**Fig. 1:**
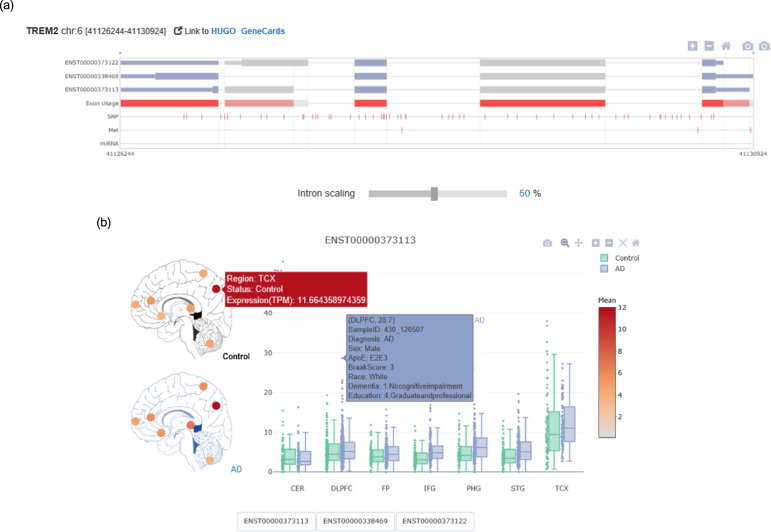
AS transcript navigator and visualizing transcript expression distribution across the seven brain regions. **a** The AS transcript navigator visualizes splicing of the transcripts of a given gene and the positions of SNPs, methylation, and miRNAs. Intron scaling allows adjusting relative intron and exon lengths. **b** Region-specific expression levels of a given transcript in normal and AD groups are visualized through a brain image heatmap (left) and a boxplot (right). In the heatmap, color is based on the average TPM value of the brain region, and hovering over each point shows detailed information. In the boxplot, each point indicates an individual, which can be hovered over to display corresponding clinical information.

**Fig. 2:**
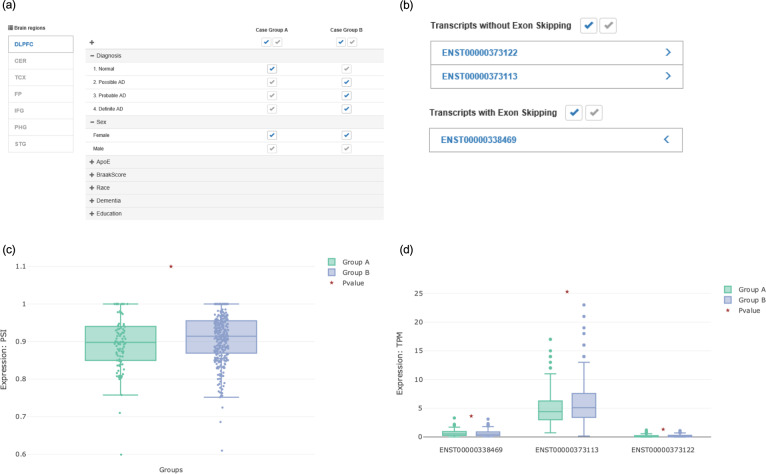
Selection of brain site and clinical features to define groups, and representative summaries of statistical results. **a** Users can select their brain region of interest (left side) and set the clinical features defining each group. In the example shown, the selected options set the analysis to compare expression between normal (Group A) and AD cases (Group B) using only females and the DLPFC region. **b** Users can also explore their transcripts of interest. In the case shown, ENST00000373122 and ENST00000373113 are considered transcripts without exon skipping, and ENST00000338469 is selected as having exon skipping. **c**, **d** *ADAS*-viewer can also explore exon (**c**) and transcript (**d**) expression in the context of AD. PSI values are estimated by calculating the ratio of transcripts without exon skipping (i.e., ENST00000373122 and ENST00000373113) to those with exon skipping (i.e., ENST00000338469), and then visualized as a boxplot (**c**). Transcript expression can be likewise analyzed and shown as a boxplot (**d**).

*ADAS*-viewer is currently available at http://genomics.chpc.utah.edu/AD, and a tutorial is available at http://genomics.chpc.utah.edu/AD/manual/Manual.pdf. *ADAS*-viewer accepts as input a variety of identifiers, such as the HUGO official gene symbol, Ensembl gene id, DNA methylation cgid, miRNA id, and SNP rsid. Given a single gene as input, *ADAS*-viewer can be used to visualize the transcripts, SNPs, methylation sites, and miRNAs annotated to that gene (AS transcript navigator, Fig. 1a), and also provides a comprehensive summary of transcript expression per gene across the seven brain regions for both normal and Alzheimer’s disease patient groups (Fig. 1b). The second aspect of *ADAS*-viewer is its analysis module, which is composed of two parts, the analysis options and the visualization of the statistical results. When performing an analysis, the user can select specific transcripts, brain regions, and cases based on clinical information (Fig. 2a and b). The selected options then generate two groups that allow the investigation of correlations between differential alternative splicing and the selected phenotype (e.g. difference by gender or ethnic background). In a further analysis step, each data type (SNP, methylation status, and miRNA expression) is co-analyzed with alternative splicing. The statistical results are displayed in plots (i.e., box and regression) along with their associated *p*-values.

### AS transcript navigator function for visualizing AS patterns

*ADAS*-viewer (Fig. 1a) illustrates all transcript isoforms, methylation sites, SNPs, and miRNA target sites within the selected gene. As in *CAS*-viewer^21^, which we previously developed, the gene is defined as the transcribed region based on Ensembl gene annotation (GTF of GRCh37.75). In the last transcript track, “Exon Usage” indicates representative exons, which are defined by clustering overlapped exons according to the various transcript isoforms and taking the longest exon in each cluster. The intron scaling bar in the navigator allows seamless transit between the genome browser displaying un-spliced pre-mRNA and the transcript viewer showing spliced mature transcripts. Namely, when intron scale is 0%, only exons are shown to allow easier visualization of splicing features, while a value of 100% is equivalent to the genome browser, depicting full-length introns. As introns are usually much longer than exons and the viewing window of the genome browser is limited, adjusting the scale helps users readily recognize AS events or splicing sites.

### Option function for setting analysis criteria

*ADAS*-viewer provides users with four options for setting criteria according to their interests (Fig. 2). These are: (1) select one brain region among the seven available, (2) select alternative splicing events at the transcript level or exon level, (3) select a clinical feature by which to define two sample groups (Fig. 2a), and finally (4) select expression at the transcript level (TPM, transcripts per million) or exon level (PSI) (Fig. 2c and d). In particular, *ADAS*-viewer allows the separation of two groups based on clinical features such as sex, Braak Score, ethnicity, education level, dementia status, ApoE status, disease status, and more. Details of the clinical information available for each brain region are described in Supplementary Table S1.

### Correlation with SNP genotype, methylation status, and miRNA target sites

*ADAS*-viewer presents statistical results in terms of four analysis components: “Comparisons”, “SNP”, “Methylation”, and “miRNA”. That is, users can first identify differential expression between two groups, then analyze the association between expression differences and transcriptional (SNPs and methylation) and post-transcriptional (miRNA) regulatory factors. The SNP analysis identifies eQTL and sQTL by combining transcript/exon expression with SNP genotype, while the methylation and miRNA analyses perform linear regression to correlate transcript/exon expression with methylation status or miRNA expression, respectively.

### Quick start

**Fig. 3:**
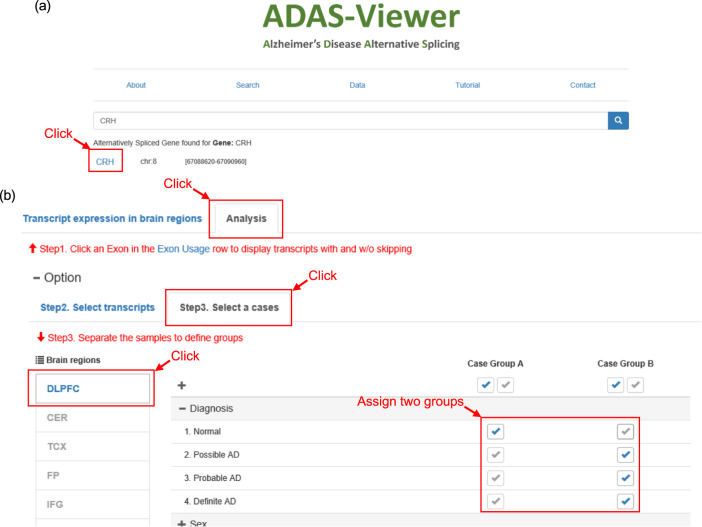
Search page and Analysis tab of *ADAS*-viewer. **a** This example searches for the gene *CRH*, which can be further investigated by clicking the result hyperlink. **b** In the Analysis tab, users may compare data between groups, in this example between normal cases (Group A) and AD cases (Group B).

We present a sample analysis to demonstrate how a user can easily explore the *ADAS*-viewer application and conduct an analysis. First, the user searches on a gene of interest using the Search page of *ADAS*-viewer (Fig. 3a). Next, the user sets options on the “Analysis” tab—i.e., the brain region and clinical features to consider (Fig. 3b). For the purpose of this example scenario, we search on the gene symbol *CRH*, select the “DLPFC” brain region, and assign groups based on disease status (“Normal” and “AD”). We then use the “Transcript Expression” mode to perform analysis with three different components (see Methods).

**Fig. 4:**
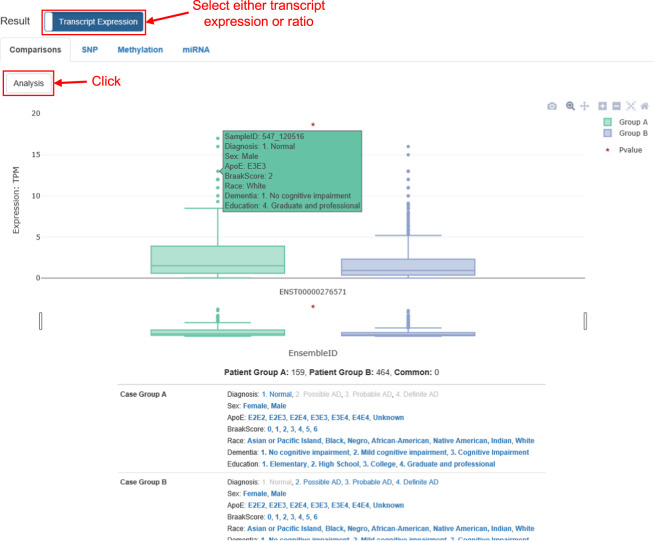
Comparisons tab of *ADAS-viewer* “Transcript Expression” analysis results. This example compares expression of ENST00000276571 between normal and AD patients.

First, to identify a transcript isoform or exon with differential expression between the user-defined groups, *ADAS*-viewer visualizes the distribution of transcript (TPM) or exon (PSI) expression. This plot is illustrated in Fig. 4, in which the x-axis corresponds to analysis groups (Normal and AD) and the y-axis to transcript expression of ENST00000276571 in the DLPFC. Throughout *ADAS*-viewer visualizations, each dot plot and boxplot respectively present detailed clinical and distribution (i.e., quantiles) information through mouse-over pop-ups. In transcriptional expression mode, it shows only outliers as dots in order to reduce computational demand. Below the plot, it presents the number of samples and selected clinical features for each group.

**Fig. 5:**
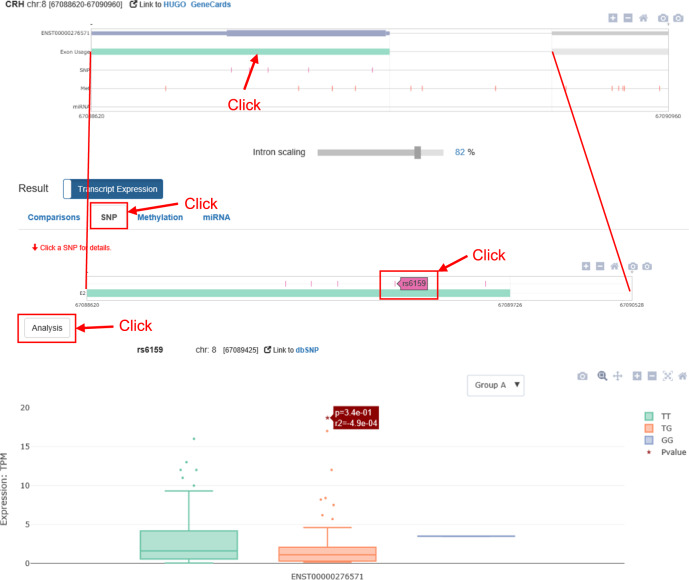
SNP tab of *ADAS-viewer* “Transcript Expression” analysis results. This example shows an association test of rs6159 with ENST00000276571 expression.

To test sQTLs: Once an exon has been selected by clicking in the AS transcript navigator, a zoomed-in view of the selected exon, including downstream and upstream flanking exons, is displayed in the SNP panel (Fig. 5). The user then selects the specific SNP they want to investigate and analyzes it via the “Analysis” button. This will generate a boxplot visualizing the distribution of transcript expression according to SNP genotype. As an example, Fig. 5 presents the result of an association test between the rs6159 polymorphism and ENST00000276571 expression in the DLPFC.

**Fig. 6:**
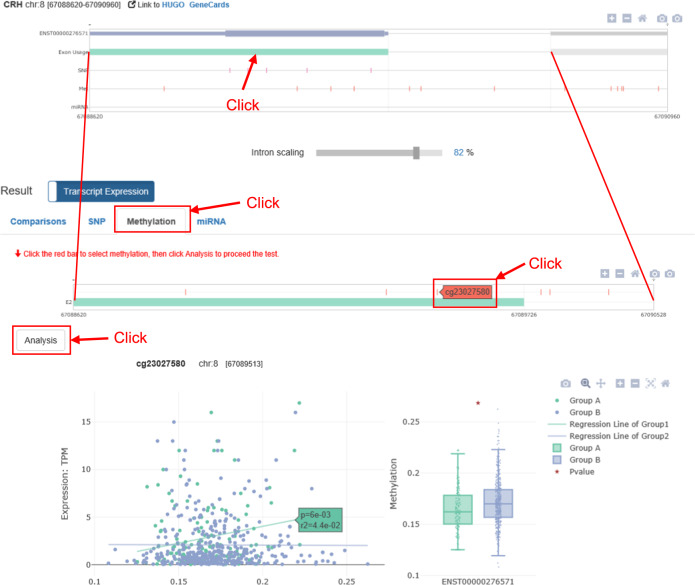
Methylation tab of *ADAS-viewer* “Transcript Expression” analysis results. This example shows an association test of cg23027580 methylation levels with ENST00000276571 expression and an investigation into differential methylation between normal and AD groups.

To test epigenetic association: As in the SNP panel, the zoomed-in exon view allows the user to select a methylation probe or miRNA and explore its association with transcript expression via the “Analysis” button. As an example, Fig. 6 illustrates the result of an association test between ENST00000276571 expression and the cg23027580 methylation probe.

Moreover, users can explore the same analyses in terms of alternative splicing under the same criteria by selecting the “Transcript Ratio” mode, which uses PSI values (see Methods) where the above examples refer to transcript expression. For instance, in Supplementary Fig. 1, we show the result of comparing the splicing rate of the 2^nd^ exon of *PRPF38B* in different brain regions between AD and normal cases.

### Case study

We present a case study to demonstrate the practical utility of *ADAS*-viewer for performing discovery analyses in the given datasets.

1. Identification of an eQTL association for rs1324551 of *FAS*.
  Using *ADAS*-viewer, we found that rs1324551 has an AD-related eQTL association with transcript ENST00000355740 of the Fas cell surface death receptor (*FAS*). Namely, expression of ENST00000355740 was increased in the brains of six AD patients with rs1324551-T, specifically in the DLPFC (*p* = 7.6e−16), FP (*p* = 7.4e−10), IFG (*p* = 3.3e−7), TCX (*p* = 4.2e−2), STG (*p* = 7.4e−9), and PHG (*p* = 8.5e−6) (Fig. 7). *FAS* is a well-known AD-associated gene that modulates apoptosis and neuronal loss, leading to progression of AD neuropathology^22^. *FAS* mRNA expression is known to be upregulated in the brains of AD patients^23,24^, and dysregulation of *FAS* is significantly associated with more rapid AD progression and reduced total brain size^22,25^. Therefore, our eQTL finding may suggest that rs1324551-T in *FAS* may contribute to AD progression by increasing *FAS* mRNA levels. Furthermore, expression of *FAS* mRNA in the para-hippocampal gyrus region, which is linked to dementia, tended to increase more in AD patients with severe cognitive impairment compared to those with moderate cognitive impairment (Fig. 7h).

**Fig. 7:**
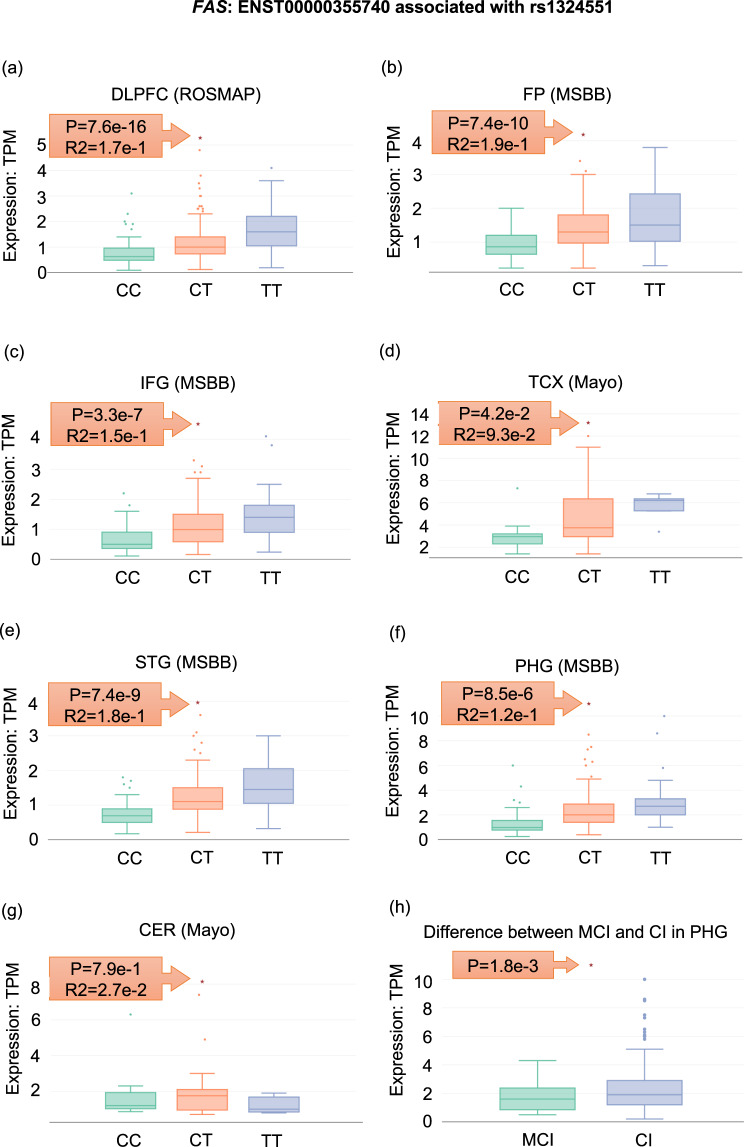
An eQTL association for rs1324551 of *FAS*. **a**–**g** Expression distribution of ENST00000355740 according to rs1324551 genotype in seven brain regions of AD patients. **h** Differential expression of ENST00000355740 in the PHG between AD patients with mild cognitive impairment (MCI) and severe cognitive impairment (CI).
2. Identification of Braak stage-dependent transcript expression of *PSENEN* in AD

Braak staging is a method for classifying the degree of pathology in AD (Braak and Braak, 1991). Using *ADAS*-viewer, we found the expression of Presenilin enhancer 2 (*PSENEN*) transcript ENST00000222266 to be significantly down-regulated in cases with high Braak stage (5 and 6) in the DLPFC (*p* = 9.3e−3), FP (*p* = 1.2e−3), STG (*p* = 4.7e−3), and PHG (*p* = 2.3e−2). While there was no significant expression change in the IFG region, this AD-associated alteration was replicated in other regions of two different cohorts. Furthermore, previous studies have reported polymorphisms of *PSENEN* as being AD risk loci^26,27^.

## Discussion

*ADAS*-viewer provides researchers with the ability to effectively navigate multi-dimensional genomic data from seven brain regions and three independent cohorts in order to investigate AD pathology. This web-based application has four distinct benefits as follows:

First, researchers can systemically and comprehensively explore the molecular characteristics of AD patients in specific brain regions as well as replicate the results using multi-omics data and independent cohorts. For example, we demonstrated the utilization of multi-omics data through investigating one gene, Corticotropin releasing hormone (*CRH*) (See the Quick Start in the Results, Figs. 3–6). Furthermore, the included datasets provide not only systemic information across the brain, but also potentially allow for replicating results based on clustering the frontal region (DLPFC; ROSMAP, FP; MSBB, and IFG; MSBB) and the temporal region (TCX, Mayo Clinic; STG, MSBB; PHG, MSBB), although these areas are not exactly the same in terms of molecular profiles. Figure 7 shows an eQTL in the *FAS* gene that is coincidently significant in all brain regions except CER, which region is known to not be relevant for AD. In addition, *ADAS*-viewer enables performing brain region-specific studies in the context of of AD. Finally, *ADAS*-viewer allows users to identify potential AD-specific alterations in the CER and TCX regions through comparison with PSP or pathogenic aging cases.

Second, the clinical information incorporated into *ADAS*-viewer (see Methods) allows researchers to carry out user-defined analyses. For instance, researchers can explore comparisons between normal and AD cases with a specific sex status, thereby identifying sex-specific eQTL/sQTL, or study the biological mechanism of AD progression through comparing AD and normal cases. For example, Supplementary Fig. 2 shows differential expression of transcript ENST00000222266 of *PSENEN* between AD cases having low (1-4) and high (5-6) Braak stage. This finding and similar analyses may help researchers better understand the biological mechanisms that contribute to each degree of AD pathology. Finally, differential methylation status and its effects on transcript expression can be evaluated in the context of AD and normal cases (Fig. 6).

Third, through allowing the investigation of both transcript isoforms and AS exon expression, *ADAS*-viewer enables researchers to readily carry out integrative analysis of RNA-seq and WGS data to identify eQTL and sQTL. In fact, our case study showed a functional role of rs1324551, which can be defined as an eQTL (Fig. 7). Also, we demonstrate that skipping of the 2^nd^ exon of *PRPF38B* tends to be more frequent in AD (Supplementary Fig. 1).

Fourth, researchers can use *ADAS*-viewer to evaluate whether their own experimental results can be replicated in a given dataset. For example, if researchers identified genes with differential expression in an animal model, such as a mouse, they can easily use *ADAS*-viewer to interrogate the same genes using data from the human brain. Also, researchers investigating AD-associated SNPs identified through GWAS can use *ADAS*-viewer to explore their functional roles.

Although well-designed web-based tools to explore AD already exist, most of those tools focus on studying a single layer of omics data, and furthermore only support visualizing analysis results based on set criteria such as AD vs. normal patients, or within a single brain region. For example, AlzRisk^28^ is a database that includes epidemiologic reports and evaluates epigenetic facts. The tool exQTLServe^29^ does integrate multi-omics data, including RNA-seq, SNPs, and methylation, but focuses on performing association tests between transcriptional expression and SNPs or methylation in only one brain region. Meanwhile, the Allen Brain Atlas^30^ incorporates multiple brain regions, but only for visualizing heatmaps of transcriptional expression. Our implementation of *ADAS*-viewer thus provides an innovative, user-friendly platform with which researchers can explore publicly available multi-omics data and flexibly perform statistical analyses across diverse brain regions in the context of AD. *ADAS*-viewer will be a useful resource for testing molecular biomarkers in AD and for carrying out integrative analyses to gain a comprehensive understanding of molecular data specific to AD pathology.

While there are many advantages to *ADAS*-viewer, as described here, it is worth pointing out some limitations to avoid over-estimation of this tool. First, although *ADAS*-viewer includes RNA-seq and WGS data for all seven regions, methylation and miRNA data are currently available only for the DLPFC region and only from ROSMAP. This might change after additional data from Mayo Clinic and MSBB become publicly available. Second, other neurologic diseases such as PSP and pathologic aging can currently only be explored in the context of the CER and TCX regions and only in samples from Mayo Clinic. Third, comprehensive clinical information is not provided for the CER and TCX region data generated by Mayo Clinic; only disease status, sex, and ApoE status are available. Fourth, the bioinformatics and statistical methods applied to data analyses in *ADAS*-viewer are not novel, but they are well-developed methods that are widely accepted.

In conclusion, we propose a web-based and publicly available application that enables to comprehensively analyze multi-omics data in various brain regions obtained from independent studies. We present the potential discovery utility of *ADAS-viewer* in potential identification of biomarker underlying AD progression in which *ADAS-viewer* can function to intuitively visualize and comprehensively analyze multi-omics data.

## Methods

### Data

#### RNA-seq data

We downloaded RNA-seq data (BAM files) collected across seven brain regions (CER, TCX, DLPFC, FP, IFG, PHG, and STG) from the Synapse database (www.synapse.org, accessed on 10 April 2019). The data were generated from three independent cohorts in the Accelerating Medicines Partnership-Alzheimer’s Disease (AMP-AD) project: Mayo Clinic (synapse id, CER: syn5049298 and TCX: syn3163039), the ROS and MAP (ROSMAP) studies (syn3388564), and the Mount Sinai Brain Bank (MSBB) (syn7416949). Detailed information on each brain region and individual demographics are given in Supplementary Table S1. To remove biases originating from the use of different reference versions and mapping parameters for each respective cohort, we converted each mapped BAM file into a FASTQ file using samtools v.1.9^31^, and then re-mapped the converted FASTQ files onto the hg19 human reference genome using STAR aligner v.2.5^32^. For our gene model (i.e., exon, intron, and transcript information), we used a GTF file obtained from Ensembl (GRCh37.75 based on hg 19, ftp://ftp.ensembl.org/pub/release-75/gtf/homo_sapiens, accessed on Oct. 13, 2018). We estimated transcript expression in terms of TPM using RSEM v.1.3.0^33^.

#### Whole genome sequencing (WGS) data

We obtained the whole-genome sequencing (WGS) data as per-chromosome VCF files generated from a joint analysis of the three cohorts (synapse id: syn11707419, accessed on 10 June 2019); these files included variants called across the cohorts. The raw WGS data was mapped to the GRCh37 human reference using Burrows-Wheeler Aligner (BWA-MEM v0.7.8), duplicate reads were marked using Picard tools v1.83, and the data processed through the steps of local realignment around indels, base quality score recalibration (BQSR), and variant calling using the Genome Analysis Toolkit (GATK v3.4.0).

#### Methylation and miRNA data

We downloaded methylation and miRNA data collected from the DLPFC region in the ROSMAP cohort (synapse id: syn3387325, accessed on 10 April 2019), which were generated using the Illumina InfiniumHumanMethylation450 bead chip assay and the NanoString nCounter miRNA expression assay, respectively. The methylation dataset was adjusted for age, sex, and experimental batch, and the miRNA dataset was normalized and corrected by Combat with the cartridges as batches.

#### miRNA target sites

We complied miRNA target sites in the 3’ UTRs of genes by integrating target databases generated from two different methods, including an experimentally validated and computationally predicted dataset we have previously published^34^; these databases were (1) miRTarBase^35^, including miRNA-target interactions experimentally validated via reporter assay and western blots; (2) TargetScan (Release 7.0)^36^, sites computationally predicted through conserved complementarity; and (3) MicroRNA.org^37^, sites computationally predicted using the miRanda algorithm. To reduce false positive target predictions, we first obtained all relations between miRNAs and mRNAs documented in miRTarBase. Then, we defined the targeted genomic coordinates for each relation using TargetScan and MicroRNA.org. Finally, we selected those miRNA target sites occurring within 3’UTR regions according to the Ensembl reference annotation (GRCh37.75). This process yielded 402,676 unique miRNA-target pairs associated with 1,388 miRNAs and 9,720 genes.

#### Clinical information

We obtained clinical information for the three cohorts included in this study: (1) Mayo Clinic: syn5049298 and syn3163039, (2) ROSMAP: syn3157322, and (3) MSBB: syn7392158. Users of *ADAS*-viewer may divide these datasets for inter-group comparisons or set criteria to analyze the data based on sex, Braak score, ethnicity, education level, dementia status, ApoE status, disease status, and more. Details on the available information are given in Table S1.

Specifics on how many samples from each omics dataset are included in *ADAS*-viewer are available at http://genomics.chpc.utah.edu/AD/manual/Data.pdf.

### Statistical methods

#### Calculation of PSI expression ratio between two groups of transcripts

*ADAS*-viewer provides for analyses on the basis of either transcript expression (as TPM) or exon expression (as percent spliced in, PSI) in the context of AS events such as exon skipping, intron retention, alternative 5′ splice site use, and alternative 3′ splice site use. PSI values can be calculated using TPM values, and are estimated in a three-step process: (1) separating transcripts of a single gene into two groups: one with the splicing event and the other without; (2) summarizing expression (TPM) for each group, and (3) taking the ratio of the sums of the two groups as the PSI value. In other words, PSI (ψ_t_) is estimated according to the equation proposed by IVAS^38^, a Bioconductor package for investigating alternative splicing, as follows:

$$f\left( {\uppsi {\mathrm{t}}} \right) = \mathop {\sum}\limits_{i = 1}^n {\left( {X_i} \right)} /\left(\mathop {\sum}\limits_{s = 1}^n {\left( {X_s} \right)} + \mathop {\sum}\limits_{i = 1}^n {\left( {X_i} \right)} \right)$$

where Xi and Xs indicate the transcript group with and without the alternatively spliced exon, respectively. *ADAS*-viewer uses this PSI value to perform analyses using WGS, methylation, and miRNA data.

#### Identification of differentially expressed transcripts and exon

*ADAS*-viewer enables users to identify transcripts or exons that are differentially expressed across seven brain regions between two user-specified groups defined based on clinical information such as sex, age, ethnicity, or disease status. The significance of the between-group difference is evaluated using Welch’s two-sample *t*-test.

#### Identification of genetic elements associated with expressions

*ADAS*-viewer provides functions for investigating the association of genetic variants with expression of transcripts or exons in the seven included brain regions through the integration of WGS and RNA-seq data. In other words, users can use *ADAS*-viewer to identify expression quantitative trait loci (eQTL) and splicing quantitative trait loci (sQTL). Such associations are identified by performing a linear regression test between transcript or exon expression (i.e., TPM or PSI) and the genotype of a given SNP.

#### Identification of epigenetic elements associated with expressions

*ADAS*-viewer applies linear regression to correlate expression (i.e., TPM or PSI) with methylation level or miRNA expression, thereby identifying transcriptome-associated epigenetic factors. The significance of these correlations is evaluated using Welch’s two-sample *t*-test.

## Supplementary information

SUPPLEMENTAL MATERIAL

## Data Availability

Mayo clinic dataset: https://www.synapse.org/#!Synapse:syn5550404^18^ ROSMAP dataset: https://www.synapse.org/#!Synapse:syn3219045^19^ MSBB dataset: https://www.synapse.org/#!Synapse:syn3159438^20^ miRTarBase: http://mirtarbase.cuhk.edu.cn/php/index.php^35^ TargetScan: http://www.targetscan.org/vert_72^36^ MicroRNA.org: http://www.microrna.org^37^

## Code availability

The html, java script, and R codes are available at Github (https://github.com/younghee93/ADAS).
