# Supplementary material for: ADAS-viewer: web-based application for integrative analysis of multi-omics data in Alzheimer’s disease
Source: NPJ Syst Biol Appl. 2021 Mar 19;7:18. doi: 10.1038/s41540-021-00177-7 (PMC7979890; doi:10.1038/s41540-021-00177-7)
Supplement: Supplementary file 1 — SUPPLEMENTAL MATERIAL [file 41540_2021_177_MOESM1_ESM.pdf]

Supplementary Material

***ADAS*-viewer: web-based application for integrative analysis of multi-omics data in Alzheimer's disease**

Seonggyun Han [1], Jaehang Shin [2], Hyeim Jung [1], Jane Ryu [1], Habtamu Minassie [2], Kwangsik Nho [3,4], Insong Koh [5], and Younghee Lee [1,2,*]

[1]Department of Biomedical Informatics, University of Utah School of Medicine, Salt Lake City, UT, US. [2]Department of Biomedical Informatics, University of Utah Asia campus, Incheon, South Korea. [3]Center for Computational Biology and Bioinformatics, Indiana University School of Medicine, Indianapolis, IN, US. [4]Department of Radiology and Imaging Sciences and Indiana Alzheimer Disease Center, Indiana University School of Medicine, Indianapolis, IN, US. [5]Department of Physiology, Hanyang University, Seoul, South Korea.

Corresponding Authors: Younghee Lee, Ph.D.

Corresponding authors' address: Younghee Lee

Department of Biomedical Informatics,

University of Utah School of Medicine,

Salt Lake City, Utah, USA,

younghee.lee@utah.edu

***PRPF38B*: exon skipping of exon 2**

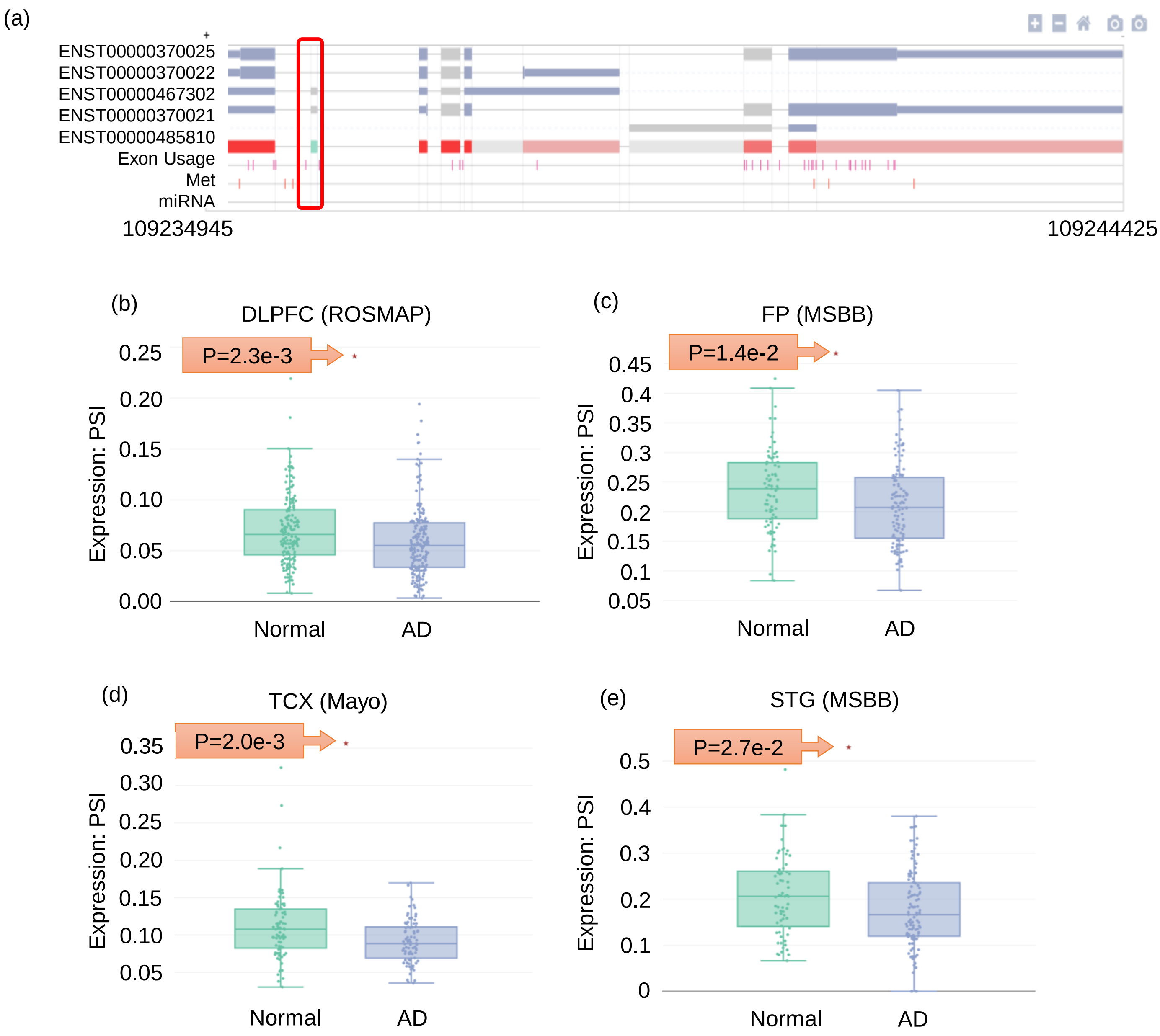

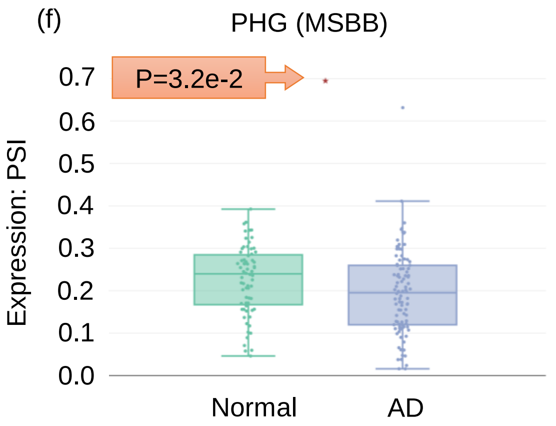

Supplementary Figure 1. (a) AS transcript navigator view of *PRPF38B*. The red box indicates exon 2. Intron scaling is set at 50%. (b-f) PSI values for each of five brain regions, referring to the inclusion ratio (see Methods) of exon 2 of *PRPF38B*.

PSENEN: ENST00000222266 associated with Braak stage

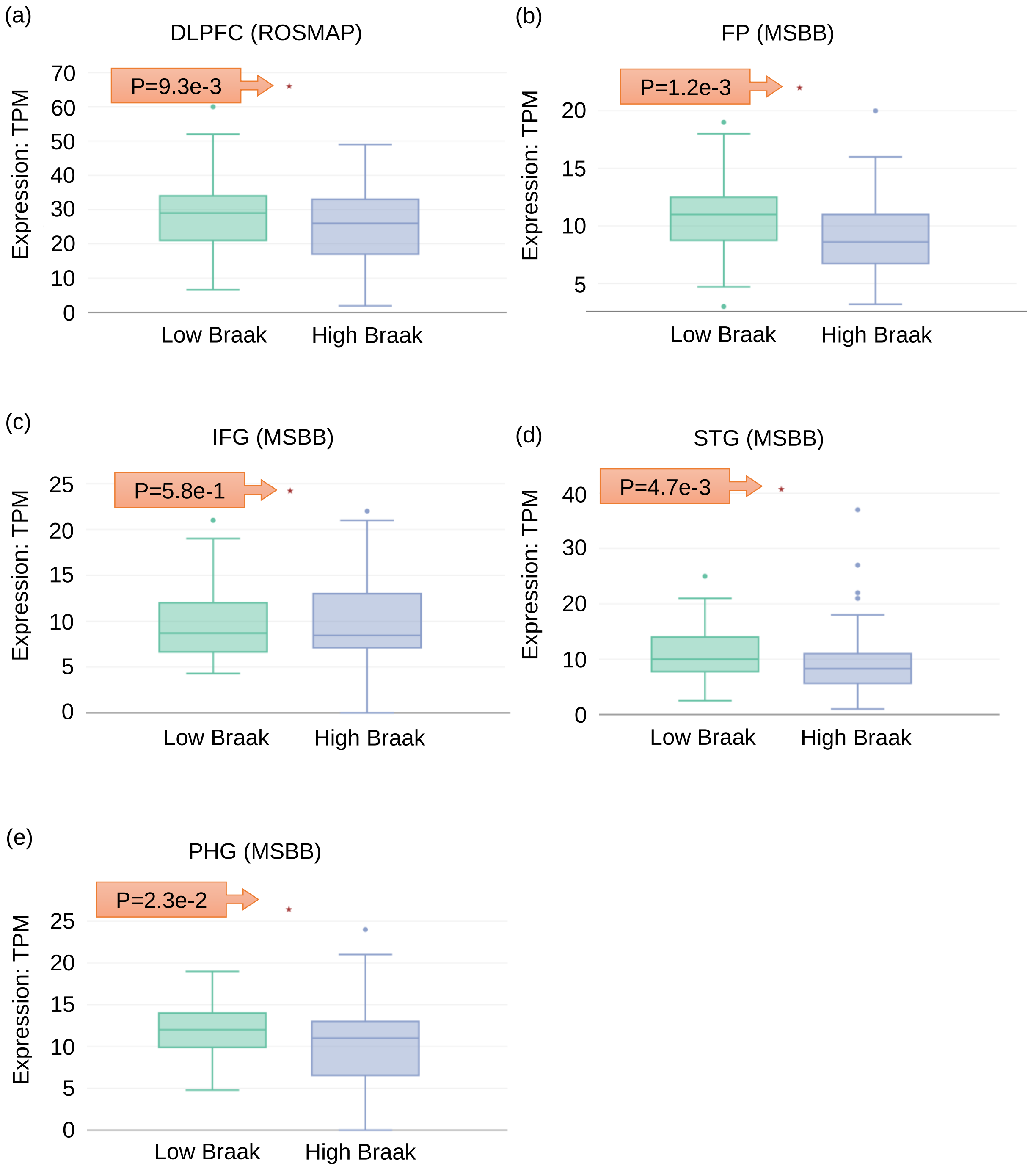

Supplementary Figure 2. Relationship between ENST00000222266 (*PSENEN*) expression and Braak stage in AD cases of each of five brain regions. The x-axis and y-axis refer Braak stage groups (i.e., low Braak group (1-4) and high Braak group (5-6) and TPM value, respectively.
